# Rapid *in Vitro* Quantification of *S. aureus* Biofilms on Vascular Graft Surfaces

**DOI:** 10.3389/fmicb.2017.02333

**Published:** 2017-12-05

**Authors:** Monika Herten, Theodosios Bisdas, Dennis Knaack, Karsten Becker, Nani Osada, Giovanni B. Torsello, Evgeny A. Idelevich

**Affiliations:** ^1^Clinic for Vascular and Endovascular Surgery, University Hospital Münster, Münster, Germany; ^2^Department of Vascular Surgery, St. Franziskus-Hospital Münster, Münster, Germany; ^3^Institute of Medical Microbiology, University Hospital Münster, Münster, Germany

**Keywords:** antimicrobial activity, ATP assay, biofilm quantification, colony-forming units (CFU), crystal violet staining (Cry), vascular graft

## Abstract

**Objectives:** Increasing resistance of microorganisms and particularly tolerance of bacterial biofilms against antibiotics require the need for alternative antimicrobial substances. *S. aureus* is the most frequent pathogen causing vascular graft infections. In order to evaluate the antimicrobial efficacy, quantification of the bacterial biofilms is necessary. Aim of the present study was the validation of an *in vitro* model for quantification of bacterial biofilm on vascular graft surfaces using three different assays.

**Methods:** Standardized discs of vascular graft material (Dacron or PTFE) or polystyrene (PS) as control surface with 0.25 cm^2^ surface area were inoculated with 10^−3^ diluted overnight culture of three biofilm-producing *S. aureus* isolates (BEB-029, BEB-295, SH1000) in 96-well PS culture plates. After incubation for 4 and 18 h, the biofilm was determined by three different methods: (a) mitochondrial ATP concentration as measure of bacterial viability (ATP), (b) crystal violet staining (Cry), and (c) vital cell count by calculation of colony-forming units (CFU). The experiments were performed three times. Quadruplicates were used for each isolate, time point, and method. In parallel, bacterial biofilms were documented via scanning electron microscopy.

**Results:** All three methods could quantify biofilms on the PS control. Time needed was 0:40, 13:10, and 14:30 h for ATP, Cry, and CFU, respectively. The Cry assay could not be used for vascular graft surfaces due to high unspecific background staining. However, ATP assay and CFU count showed comparable results on vascular graft material and control. The correlations between ATP and CFU assay differed according to the surface and incubation time and were significant only after 4 h on Dacron (BEB-029, *p* = 0.013) and on PS (BEB-029, *p* < 0.001). Between ATP and Cry assay on PS, a significant correlation could be detected after 4 h (BEB-295, *p* = 0.027) and after 18 h (all three strains, *p* < 0.026). The reproducibility of the ATP-assay presented as inter-assay-variance of 2.1 and as intra-assay variance of 8.1 on polystyrene.

**Conclusion:** The *in-vitro* model reproducibly quantifies biofilm on standardized vascular graft surfaces with ATP assay as detection system. The ATP assay allows accelerated microbial quantification, however the correlation with the CFU assay may be strain- and surface-dependent.

## Introduction

Although bacterial biofilms are naturally occurring in dental plaque (Sanz et al., 2017), their presence is mainly associated to critical medical conditions such as infections of the upper respiratory or urogenital tract, peritonitis, and on implanted medical devices, which are increasingly used (Lynch and Robertson, 2008; Romling et al., 2014).

Vascular grafts are used in aortic reconstructions and for lack of autologous vein material also in peripheral bypass operations. Despite highest hygienic precautionary measures during surgery, vascular graft infections (VGIs) occur in 2–4% of cases and are associated with high morbidity and mortality rates (10–75%) (Hepp, 2009; Young et al., 2012). Gram-positive microorganisms such as *Staphylococcus aureus* and *Staphylococcus epidermidis* are typically the cause of aortic graft infections (Chaufour et al., 2017).

Bacterial colonization of the vascular graft surface is associated with bacterial biofilm production, the accumulation of extracellular polymer substance matrix (EPS), consisting of polysaccharides, proteins, glycolipids, and bacterial DNA (Becker et al., 2014). The EPS shields the bacteria by preventing the penetration of macromolecules like antibiotics and inflammatory cells of the immune defense into the biofilm matrix as well as by displaying a diffusion barrier for molecules with antimicrobial properties (Olsen, 2015). Within the biofilm, subpopulations can become metabolic inactive and form dormant persister cells. Since some antibiotics are only effective for metabolic active bacteria, the resting bacteria may be insensitive toward antibiotics (Lewis, 2007).

Next to standardized pre- and peri-surgical prevention measures against VGIs (pre-screening for multidrug resistant organisms, control of infection parameters, antimicrobial prophylaxis), also regional antibiotic release from vascular grafts (pre-soaking of the graft with antibiotics) could play an effective role (Keeling et al., 2009; Kuehn et al., 2010; Bisdas et al., 2012).

Growing resistance and tolerance of bacterial biofilms against antibiotics require the need for alternative antimicrobial substances. An alternative for therapy of *S. aureus* biofilms on vascular grafts could be for example bacteriophage endolysins, such as HY-133, with specific efficacy against *S. aureus* (Idelevich et al., 2011, 2016; Herten et al., 2017). In order to evaluate the antimicrobial efficacy of such alternative antimicrobial substances, the quantification of the bacterial biofilms is necessary.

Several methods are available for the detection of bacterial biofilms, either based on (a) the accumulation of dye/pigments binding to negative charges present at the EPS, e.g., crystal violet (O'toole et al., 2000; Stepanovic et al., 2000) or safranin (Patterson et al., 2010), or (b) DNA binding dyes (DAPI, Hoechst, SYTO9 or acridine orange, Palestrant et al., 2004; Peeters et al., 2008) or qPCR, or (c) cultural method [colony forming units (CFU) calculation (Alt et al., 2004)], time to regrowth to a specific turbidity], or (d) detecting the metabolic activity [ATP (Gracia et al., 1999; Kapoor and Yadav, 2010), tetrazolium salt MTT, resazurin (Alamar blue) (Hatzinger et al., 2003; Pettit et al., 2009)] or membrane integrity (e.g., live/dead staining SYTO9/propidium iodide, Tawakoli et al., 2013).

However, quantifications of bacterial biofilms were mainly performed on polystyrene surface, only few methods are available for other surfaces (Pitts et al., 2003). For the quantification of biofilms on indwelling devices, the CFU count seems to be the standard method until now and replaced the past radioactive labeling of the bacteria (Schmitt et al., 1986).

Aim of the present study was the validation of an *in vitro* model for rapid quantification of bacterial biofilm adherence on vascular graft material with an ATP assay compared to CFU count and crystal violet (Cry) assay.

## Methods

### Overnight culture of bacteria

Two clinical biofilm-producing *S. aureus* (BEB-029 and BEB-295) isolates as well as laboratory strain SH1000 were used for experiments. Species affiliation was confirmed by detection of the *S. aureus*-specific *nuc* gene and methicillin resistance was determined by *mecA* gene detection as described elsewhere (Becker et al., 2006). The isolates were incubated overnight in tryptic soy broth (TSB) at 36C°.

### Experiment set up

**Figure 1:**
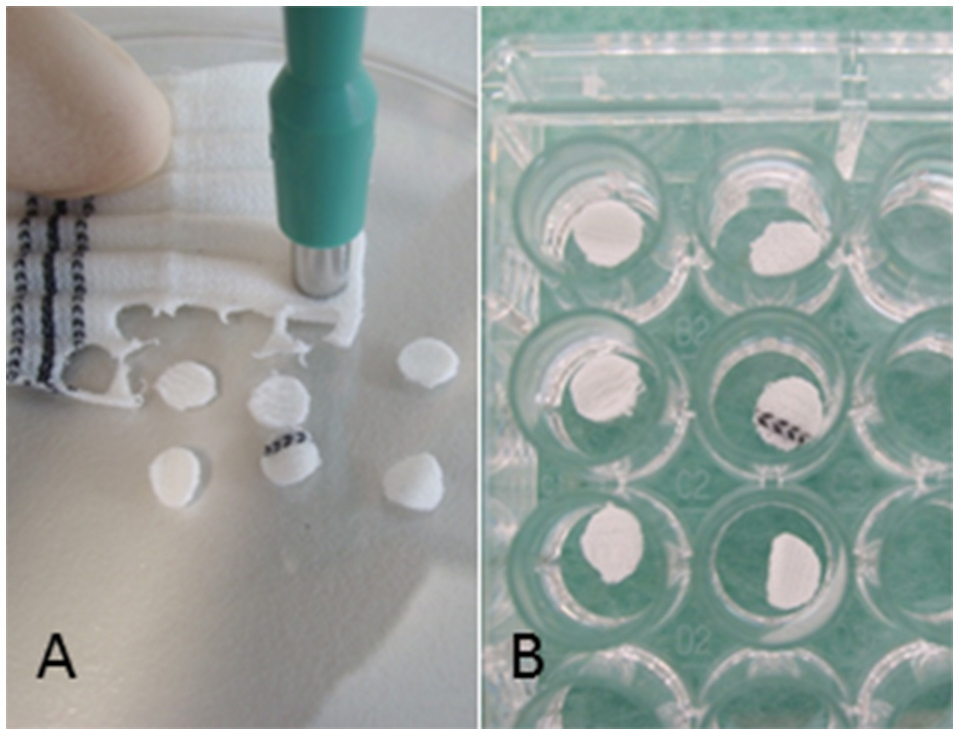
Experiment set-up: **(A)** Vascular graft material was dissected with biopsy punches resulting in a standardized surface area of 0.25 cm^2^ and **(B)** placed into 96-well polystyrene (PS) cell culture plates.

Vascular graft material (Dacron (polyester), knitted, (Uni-Graft^®^ K DV, B. Braun, Melsungen, Germany and PTFE MAXIFLO™ Ultrathin, Vascutek, Terumo, Frankfurt, Germany) were dissected with biopsy punches (ø 5 mm, pmf medical AG, Cologne, Germany) in order to yield a standardized surface area of 0.25 cm^2^ (Figure 1), The discs were positioned into 96-well polystyrene (PS) culture plates and submerged using peg lids (MBEC Assay, St. Edmonton, Canada). Polystyrene without graft material discs served as control surface. All 96 wells (with and without discs) were inoculated with a 10^−3^ diluted overnight culture in TSB with 0.25% glucose. Sterility controls were additionally performed. After 4 and 18 h incubation at 36°C, the planktonic bacteria were rinsed off twice with phosphate-buffered saline (PBS). The biofilm concentration was measured on the graft disc and on the control wells. The experiments were performed three times.

### SEM documentation of bacterial biofilms

Overnight cultures were diluted 10^−3^ and vascular grafts (Dacron, PTFE) inoculated as described above. After 18 h cultivation, the discs were rinsed twice with PBS and the bacterial biofilm was fixed in 2.5% glutardialdehyde in PBS for 24 h at 8°C. Post-fixation was performed with 1% osmium tetroxide in PBS for 20 min at room temperature. After dehydration through an ascending series of ethanol ending in 100% ethanol, the samples were dried via critical point drying (Emitech, Ashford, UK/CPD 030, Bal-Tec AG, Balzers, Liechtenstein) according to the manufacturer instructions. Afterward the discs were gold-sputtered (E5100, Polaron Instruments, UK/SC0005 Bal-Tec AG, Balzers, Liechtenstein) and analyzed with a sequential electron microscope (S800, Hitachi, Düsseldorf, Germany).

### Biofilm quantification

All tests were performed in quadruplicates for each isolate (3 strains), culture time (4 and 18 h), cultivation surface (Dacron, PTFE and PS control), and assay type.

#### ATP assay

The mitochondrial adenosine triphosphate (ATP) concentration was measured using the BacTiter-Glo™ Microbial Cell Viability assay (Promega, Mannheim, Germany) as described before (Ickert et al., 2015; Herten et al., 2017). The number of viable bacteria is quantified by their amount of ATP and is directly proportional to the ATP concentration. The assay is based on the reaction of luciferase, which catalyzes the reaction of luciferin and ATP to oxyluciferin by the emission of a luminescent signal, which is measured using a multilabel plate reader (FLX800, Biotek, Bad Friedrichshall, Germany). Preceding experiments were performed with varying amounts of lysis solution in order to determine the concentration necessary to lyse the *S. aureus* cells. The optimal relation of PBS and lysis solution was 1:3. In brief, after washing, 50 μl PBS and 150 μl BacTiter-Glo™ solution was added to each well. After an incubation period of 5 min at room temperature, the luminescent signal was recorded in counts per second. Additionally, standard measurements with defined numbers of *S. aureus* was performed, and ATP standard curves were made with defined ATP-concentrations (0.1–50 μM) for each measurement. Hands-on time for the ATP assay was 0:30 min, results were available after 0:40 min.

#### Crystal violet assay

One hundred microliters of 1% crystal violet solution were added to each well of a microtiter plate containing graft discs and incubated at room temperature for 5 min. Subsequently, the graft discs were transferred into the wells of another microtiter plate to wash out the excessive amounts of crystal violet twice with 200μl PBS. After the aspiration of PBS and drying overnight, 50 μl of absolute ethanol were added, followed by shaking the plate and incubation for 10 min. After the graft discs were removed, absorbance was measured at 570 nm via a spectrophotometer. Hands-on time for the Cry assay was 1:10 h, incubation time 12 h, results were available after 13:10 h.

#### CFU count

The number of CFUs of vital bacteria was measured by plating of serial dilutions onto the solid medium. For this, the vascular grafts were rinsed with 100 μl PBS and sonicated for 10 min in order to detach and disrupt the biofilm from the graft material into a recovery medium. After removal of graft discs, the serial dilutions of well content were plated on tryptic soy agar (TSA) in triplicate. After incubation for 18 ± 2 h at 36°C the numbers of colonies were counted. Hands-on time for the CFU assay was 2:30 h, incubation time 12 h, results were available after ~14:30 h.

### Statistical analysis

The statistical analysis was performed with software IBM SPSS Statistics for Windows (release 24, Chicago, IL; USA). Categorical variables are expressed as frequency and percentage whereas continuous variables were presented as mean ± *SD*. Before statistically testing, the Kolmogorov-Smirnov test was used to analyze each continuous variable for its normal distribution. Because the samples were not normally distributed, the non-parametric tests were used. The Mann-Whitney *U*-test was used for comparison of non-parametric variables of two independent study groups. The Pearson correlation coefficient (r) was used to evaluate the relationship between two continuous variables ATP vs. CFU on polystyrene surface, on Dacron and on PTFE and while CFU vs. Cry could be only determined on polystyrene surface because of the high staining background of Cry on the vascular material. Linear regression model was used to estimate the relationship between the dependent variable ATP and the independent variable CFU or Cry. The coefficient of determination (*R*^2^) was calculated as a statistical measure of how well the regression line approximates the real data points. Differences were considered significant at *p* < 0.05. Measurement accuracy of the ATP assay was calculated as coefficient of variability (CV) by estimating inter- and intra-assay variance.

## Results

### SEM documentation of bacterial biofilms

**Figure 2:**
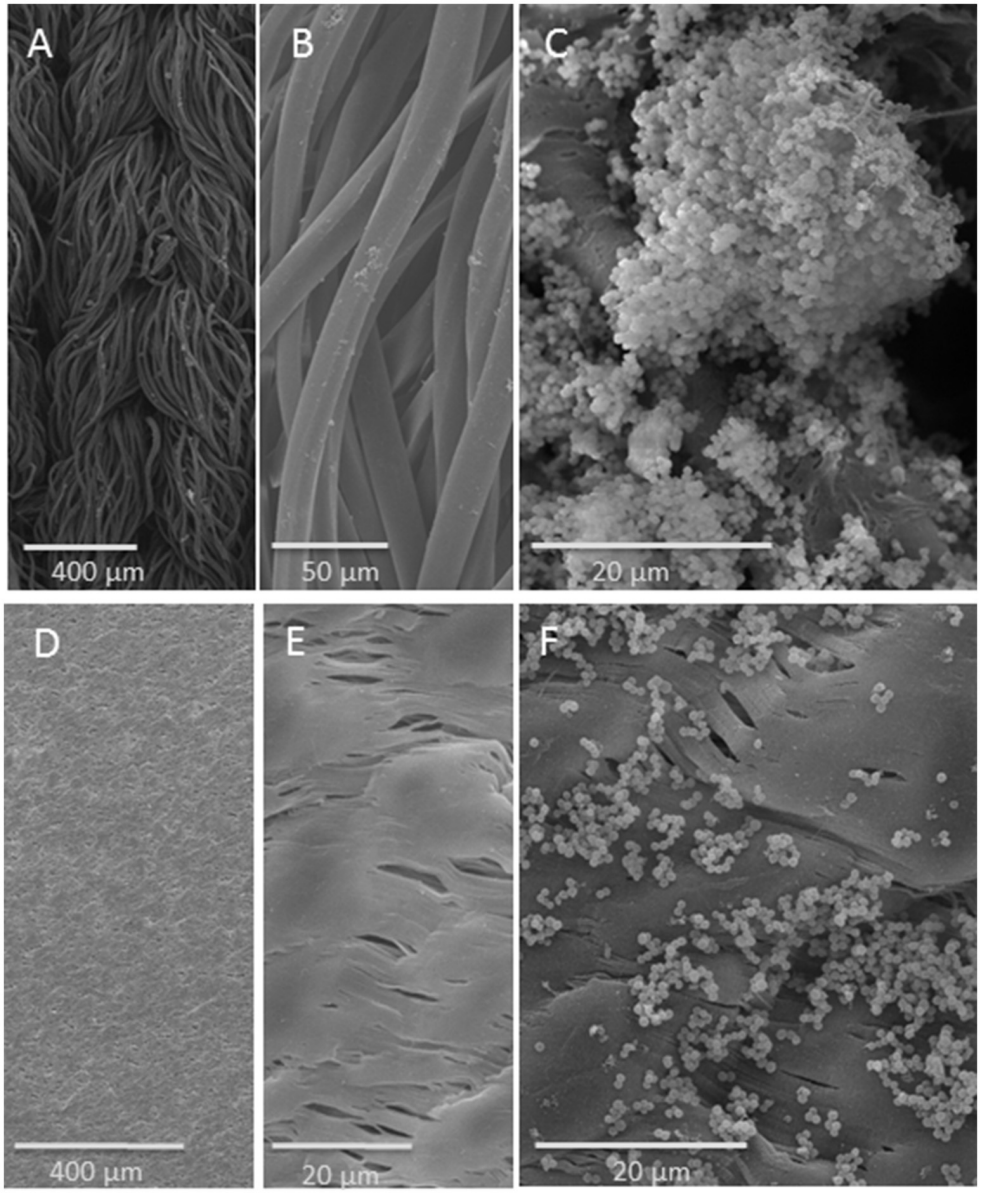
SEM analysis of vascular graft material: upper row: on Dacron fiber, lower row: on PTFE surface: **(A)** knitted structure; **(B)** fibers; **(C)** *S. aureus* strain SH1000 biofilm on Dacron fiber. **(D**,**E)** PTFE surface in different magnifications; **(F)** *S. aureus* strain SH1000 on PTFE.

SEM documentation of bacterial biofilms revealed a distinctly recognizable biofilm on the single threads of the knitted Dacron surface (Figures 2A–C). In contrast, the much smoother PTFE surface revealed accumulated bacterial groups, which were more scattered (Figures 2D–F).

### Biofilm quantification

**Figure 3:**
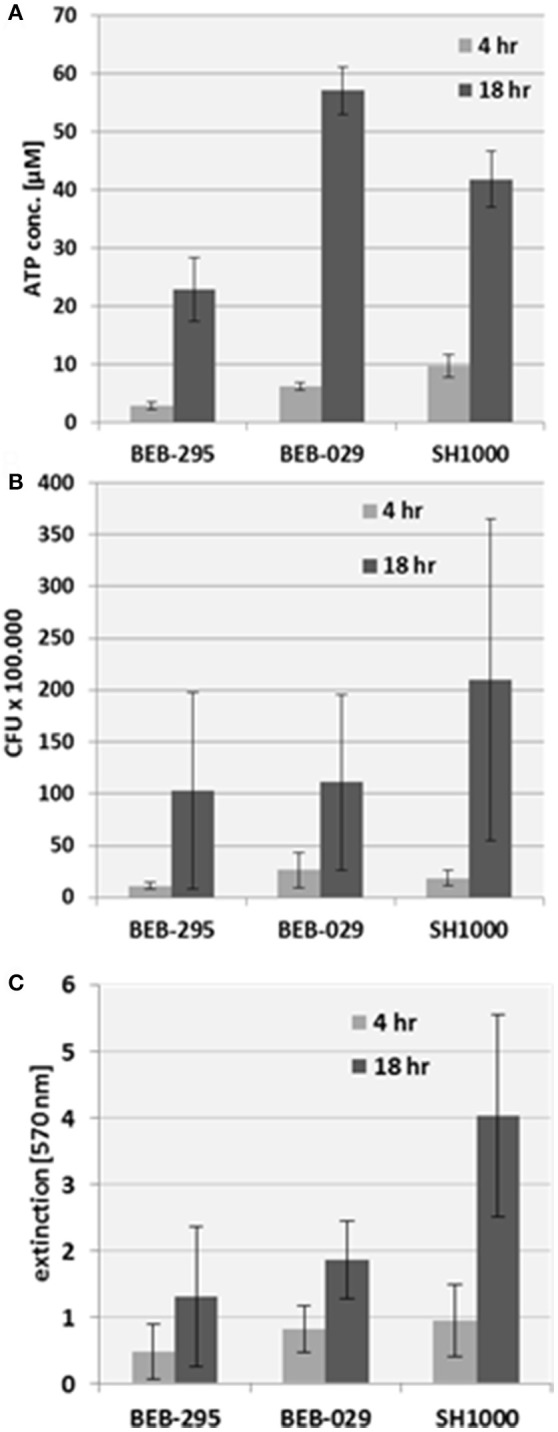
Biofilm quantification on the control surface polystyrene. **(A)** Mitochondrial ATP concentration, **(B)** CFU count, **(C)** Crystal violet staining.

All three assay methods could quantify biofilms on the PS control surface (Figure 3). The reproducibility of the ATP-assay presented an inter-assay-variance of 2.1 and an intra-assay variance of 8.1 on polystyrene. The growth pattern of the biofilm revealed an increase in bacterial biomass from 4 to 18 h incubation time in all three assay methods.

The measurement of Cry via spectrophotometry was not suitable for vascular graft surfaces because of the high unspecific background staining.

**Figure 4:**
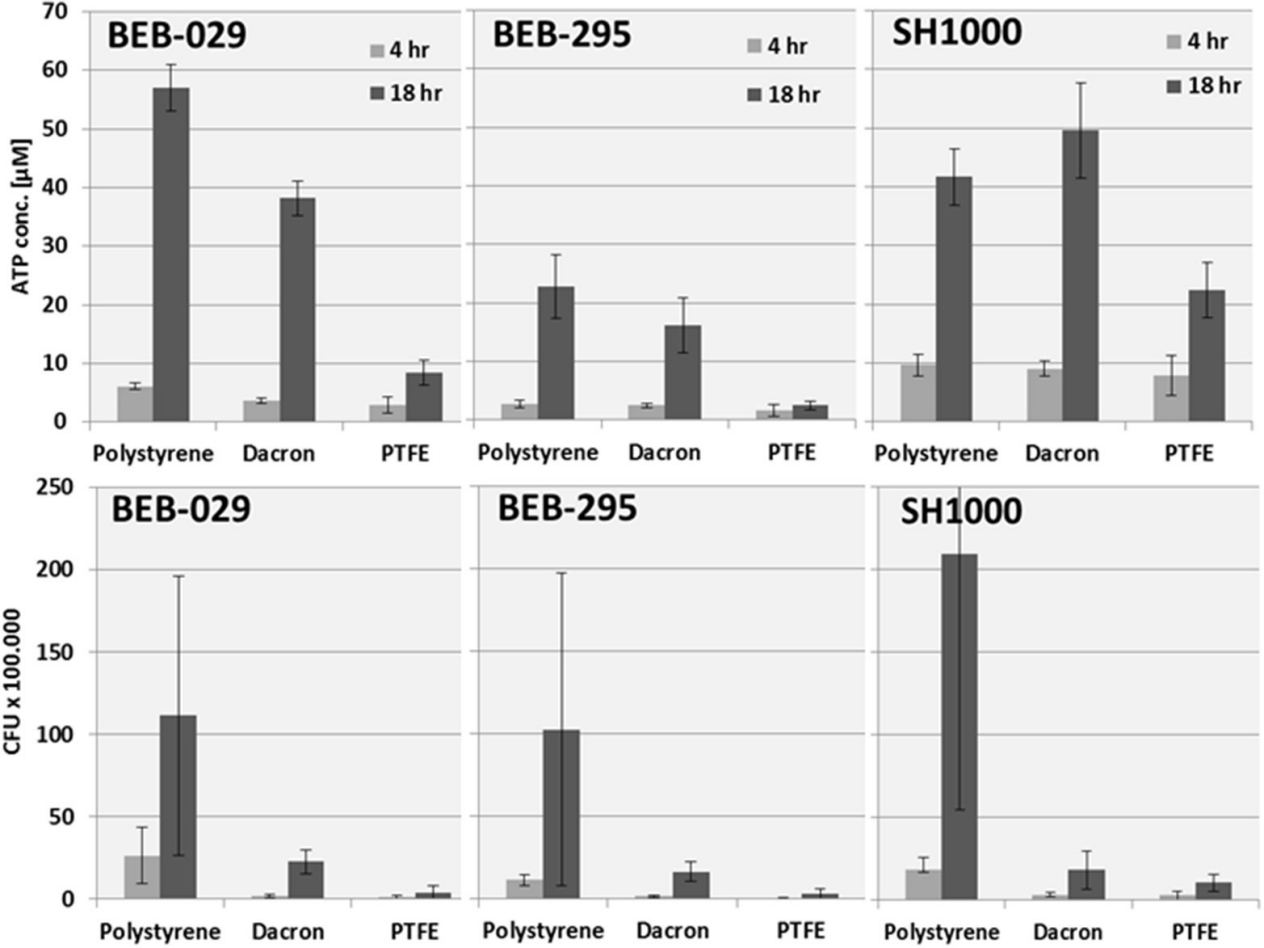
Quantification of the *S. aureus* strains on different surfaces: polystyrene, Dacron and PTFE. **Upper**: ATP concentration. **Lower**: CFU counts. Data for the polystyrene surface were additionally depicted in Figure 3.

Regarding the bacterial growth pattern on the different graft materials, *S. aureus* strain BEB-029 and BEB-295 displayed their highest ATP concentration and CFU count, respectively, on the PS control, followed by Dacron and the least on PTFE (Figure 4). This is in accordance with the growth intensity displayed on the SEM pictures. For the *S. aureus* strain SH1000, the highest ATP concentration was demonstrated on Dacron material followed by PS and PTFE, while in the CFU assay strain SH1000 showed the highest values on PS (Figure 4 lower row).

The Pearson's correlation coefficient depicted a strong correlation coefficient between ATP and CFU on Dacron after 4 h for BEB-029 (0.606, *p* = 0.013) and a moderately strong for BEB-295 (0.442, *p* = 0.086) (Table 1, Table S1). On PS a very strong correlation was detectable for BEB-029 (0.858, *p* < 0.001) after 4 h and a moderate strong one for SH1000 (0.548, *p* = 0.065) after 18 h. Likewise, a strong correlation on PS surface could be detected between ATP and Cry for BEB-295 after 4 h (0.633, *p* = 0.027), a moderately strong one for BEB-029 (0.408, *p* = 0.188) and strong to very strong correlations after 18 h for all three strains (*r* ≥ 0.741, *p* < 0.026).

**Table 1:** Pearson's correlation coefficient of the *S. aureus* strains on the different materials (Dacron or polystyrene) for ATP vs. CFU, ATP vs. Cry, and CFU vs. Cry.

**Assay**	**Material**	**Time**	**Correlation**	**Organism**
ATP vs. CFU	Dacron	4 h	Strong	BEB-029
			Moderate strong	BEB-295
		18 h	Very weak	All three strains
ATP vs. CFU	PTFE	4 h	Negative correlations	All three strains
		18 h	Negative correlations	All three strains
ATP vs. CFU	PS	4 h	Very strong	BEB-029
		18 h	Moderate strong	SH1000
ATP vs. Cry	PS	4 h	Strong	BEB-295
			Moderate strong	BEB-029
		18 h	Very strong	BEB-029
			Strong	BEB-295, SH1000
Cry vs. CFU	PS	4 h	Strong	BEB-029
			Weak	BEB-295
		18 h	Weak	BEB-029, SH1000

*The following interpretation of the correlation coefficient and the strength of relationship was applied: 0.01–0.2: very weak, 0.21–0.4 weak, 0.41–0.6 moderately strong, 0.61–0.8 strong, 0.81–1 very strong. There were no significant positive correlations between the results of the ATP vs. CFU assay on PTFE surfaces. Listed are the highest positive correlations for the time point and material used. A detailed list of all Pearson's correlation coefficient and coefficient of determination of quantification is given in the supplementary material (Table S1)*.

In contrast, there were only negative correlations between the ATP and CFU assays for the PTFE surface (range *r* = −0.224 until −0.811). The assay time until result was 0:40 h for ATP, 13:10 h for Cry, and 14:30 h for CFU assays.

Comparing CFU count with Cry assay, a strong coefficient was present only after 4 h for BEB-029 (0.628, *p* = 0.029).

## Discussion

### *In vitro* model for rapid quantification

This study evaluated an *in vitro* model for rapid quantification of bacterial biofilm adherence on vascular graft material with an ATP assay. In particular, the ATP assay was compared to the CFU count, as the gold standard method, and to the crystal violet staining. Using BEB-029, the ATP assay correlated significantly with the CFU method at 4 h where it was useful as a rapid assay for biofilm quantification. However, the correlation with CFU assay was generally strain- and surface-dependent (Table 1, Table S1). In addition, correlation between CFU and ATP assays was generally better for immature biofilm (4 h) than mature biofilm (18 h).

A comparison of ATP and Cry assay on the PS control surface revealed correlations for both biofilm types: at 4 h strong correlation for BEB-295, moderately strong ones for BEB-029 and at 18 h very strong correlation for BEB-029 and strong one for the two other strains.

Concerning the only moderately strong correlation between ATP assay and CFU count on mature biofilms on PS, it can be pointed out, that both assay systems ATP and Cry showed comparable correlations even for mature biofilms and both did not correlate with the CFU method in mature biofilm. Both assays do not require any detaching and separation of bacterial biofilm, which might be a reason for the missing correlations with the CFU in mature biofilm. Biofilm quantification revealed that the colonization of the materials used (PTFE vs. Dacron) was different over time for the three *S. aureus* biofilm-producing strains. On PTFE, the bacterial colonization was lower as determined by ATP assay and CFU counting. These quantitative results could be confirmed by qualitative visualization by SEM.

In this study, we used a static biofilm model, which delivers helpful insights in the usefulness of different assays for biofilm quantification. Dynamic biofilm models may provide more differentiated information and should be included in further investigations of vascular grafts exposed to a continuous blood flow.

### ATP assay on polystyrene (PS) surface

The ATP assay has been used previously for detection of bacterial biofilms on polystyrene (PS) surface. Gracia et al. could show that for bacterial ATP extraction from *in vitro* developed *S. aureus* biofilms on coated PS surface with high bacterial density, DMSO was found preferable in relation to commercially available bacteria lysis reagent (upper detection limits 2.3 × 10^9^ and 2 × 10^8^ CFU/ml respectively) (Gracia et al., 1999).

Kapoor et al. used the ATP bioluminescence assay for biocidal susceptibility testing of rapidly growing mycobacteria and compared it to the conventional agar plating method. They demonstrated that the ATP assay was rapid (1.5 h) and showed high sensitivity and specificity. Using this method, the ATP assay revealed a linear relationship between intracellular ATP and the cell number (CFU/ml) in the CFU range of 10^3^-10^7^ CFU/ml. (Kapoor and Yadav, 2010). In the present study, the detection limit for the ATP assay for *S. aureus* was in the range from 1.5 × 10^2^ up to more than 10^8^ cells as described in the instructions for use, thus being comparable to the detection limits described by Gracia and Kapoor (Gracia et al., 1999; Kapoor and Yadav, 2010).

Villain-Guillot et al. detected *S. epidermidis* biofilms in 96-well polystyrene microtiter plates. They presented very good correlation curves (*r* = 1.0) between the ATP-counting luminescence values and the amount of biofilm-embedded *S. epidermidis* determined by serial dilution and cultivation (CFU assay) (Villain-Guillot et al., 2007). But in contrast to our method, each biofilm was sonicated, the bacterial suspension was serially diluted and part of it was used for ATP assay and for classical plate counting. Therefore, both assays had the same diluted bacterial samples as starting material omitting any possible bias resulting from the detaching. In the present study, the ATP assay was used as rapid assay leaving out the detaching and dilutions steps. On PS surface, the correlations between ATP assay and CFU count were very strong at 4 h and moderately strong at 18 h.

### ATP assay on indwelling medical devices/biomaterials

Next to polystyrene, the ATP assay has also been applied for the detection of bacterial biofilms on indwelling medical devices/biomaterials. Gracia et al. investigated the adherence potential of different biofilm producing and non-biofilm-producing strains of *S. aureus* strains on biomaterials used in orthopedic surgery (polymethylmethacrylate, fresh bone, steel, and titanium alloys and on glass) (Gracia et al., 1997). The device material was sonicated to disintegrate bacterial clumps. They used a calibration curve of bacterial ATP vs. CFU to convert ATP moles to CFU counts. A linear relationship was found between the detected bacterial ATP and the number of bacteria (CFU/ml) in the interval between 3.5 × 10^5^ CFU/ml and 3.5 × 10^9^ CFU/ml with a high correlation (*r* = 0.99). In the present study, the ATP assay and the CFU counting method were compared displaying strong or moderate correlations on Dacron at 4 h and weak correlations at 18 h and without positive correlations on PTFE. Our data cannot be directly compared since Gracia et al. used the detached bacteria solution for the standard curve.

### Bacterial adherence to vascular grafts

Regarding bacterial adherence to vascular grafts some *in vitro* studies have been published. All of them determined bacterial adherence as the number of CFUs dislodged from graft material by sonication.

Schmitt et al. used 1 cm^2^ squares of graft material (*n* = 5/6) immersed in the bacterial suspension of *S. aureus, Escherichia coli, and S. epidermidis*. Bacterial adherence depended on Dacron, and was least on PTFE graft material (Schmitt et al., 1986). This is in line with our findings of the SEM documentation (more scattered accumulated bacterial groups on PTFE) and was confirmed by the ATP assay and the CFU count. Kuehn et al. used the same model for evaluation antibiotic pretreatment of vascular prostheses using seven grafts per group. They could show that the risk of vascular graft infection was reduced by pretreating the prostheses with antibiotics while an antibiotic/fibrin compound exhibited an effect of delayed antibiotic release (Kuehn et al., 2010). In the present study, we used standardized vascular graft surfaces cut with a biopsy punch with a defined area which might be easier than individually cut small matching squares.

Sasaki investigated the bacterial penetration of *Pseudomonas aeruginosa* from the outer surface of vascular grafts material comparing elastomer-sealed and gelatin-coated surface treatment of the outside of the graft. In the experimental setup, 12 or 18 grafts were cut into 6-cm segments, placed in a U-shaped configuration on culture plates and inoculated at the outer graft surface. Data revealed a bacterial invasion through the prosthetic graft material starting 6 h post-bacterial immersion with no significant differences between the two different treatments of the outer polyester surface (Sasaki, 2014).

It is noticeable that the group size was 5, 6, or 7 and the experiments were performed only once. This could be due to the fact that the determination of bacterial adherence via CFUs is time- and labor-consuming.

The CFU assay requires the incubation of diluted bacteria sample. Mandatory prerequisite for the dilution is the dissolving of the bacterial biofilm which may be a distinct bias of the different methods available.

Several investigators have reported inconsistent results regarding the development of infection- resistant vascular grafts. In addition, numerous antibiotics have been investigated, sometimes with different carriers to encourage a prolonged antimicrobial effect (Kuehn et al., 2010).

## Conclusion

The *in-vitro* model could be used for reproducible quantification of biofilm adhesion on standardized vascular graft surfaces with ATP assay as detection system. Compared to the CFU assay, the ATP assay allows accelerated quantification of bacteria, however the correlation with CFU assay may be strain- and surface-dependent.

## Author contributions

MH and EAI: designed this project; MH, TB, DK, KB, NO, GT, and EAI: contributed to the experiments, analysis and interpretation of the work; MH: drafted the manuscript; MH, TB, DK, KB, NO, GT, and EAI: reviewed the manuscript for intellectual content.

### Conflict of interest statement

The authors declare that the research was conducted in the absence of any commercial or financial relationships that could be construed as a potential conflict of interest.

## Supplementary material

Table S1 Pearson's correlation coefficient and coefficient of determination of quantification for all assays, surfaces, and time points.

## Abbreviations

ATP: mitochondrial ATP concentration as measure of bacterial viability
BEB-029, BEB-295, SH1000: biofilm-producing *S. aureus* isolates
CFU: colony-forming units
CV: coefficient of variability
Cry: crystal violet staining
EPS: extracellular polymer substance matrix
MTT: 3-(4,5-dimethylthiazol-2-yl)-2,5-diphenyltetrazolium bromide for assessing cell metabolic activity
PTFE: polytetrafluorethylene
*R*^2^: coefficient of determination
*r*: Pearson correlation coefficient
SEM: scanning electron microscopy
SYTO9: cell-permeant nucleic acid stains
VGI: vascular graft infections.
