# Supplementary material for: Rapid in Vitro Quantification of S. aureus Biofilms on Vascular Graft Surfaces
Source: Front Microbiol. 2017 Dec 5;8:2333. doi: 10.3389/fmicb.2017.02333 (PMC5723318; doi:10.3389/fmicb.2017.02333)
Supplement: Table S1 — Pearson's correlation coefficient and coefficient of determination of quantification for all assays, surfaces, and time points. [file DataSheet1.DOCX]

| **Assay** | **Material** | **Time** | **Organism** | **Pearson correlation coefficient** | | **Coefficient of determination** |
| --- | --- | --- | --- | --- | --- | --- |
|  |  | **[h]** |  | **r** | **p** | **R^2^** |
| **ATP vs. CFU** | **Dacron** | **4** | **BEB-029** | **0.606** | **0.013** | **0.367** |
|  |  |  | **BEB-295** | **0.442** | **0.086** | **0.196** |
|  |  |  | **SH1000** | **-0.065** | **0.840** | **0.004** |
|  |  | **18** | **BEB-029** | **0.173** | **0.523** | **0.030** |
|  |  |  | **BEB-295** | **-0.230** | **0.392** | **0.053** |
|  |  |  | **SH1000** | **0.051** | **0.874** | **0.003** |
| **ATP vs. CFU** | **PTFE** | **4** | **BEB-029** | **-0.224** | **0.422** | **0.050** |
|  |  |  | **BEB-295** | **-0.703** | **0.007** | **0.495** |
|  |  |  | **SH1000** | **-0.474** | **0.119** | **0.225** |
|  |  | **18** | **BEB-029** | **-0.315** | **0.253** | **0.099** |
|  |  |  | **BEB-295** | **-0.290** | **0.337** | **0.084** |
|  |  |  | **SH1000** | **-0.811** | **0.001** | **0.658** |
| **ATP vs. CFU** | **Polystyrene** | **4** | **BEB-029** | **0.858** | **<0.001** | **0.737** |
|  |  |  | **BEB-295** | **-0.175** | **0.586** | **0.031** |
|  |  |  | **SH1000** | **-0.363** | **0.246** | **0.132** |
|  |  | **18** | **BEB-029** | **0.077** | **0.811** | **0.006** |
|  |  |  | **BEB-295** | **-0.142** | **0.659** | **0.020** |
|  |  |  | **SH1000** | **0.548** | **0.065** | **0.301** |
| **ATP vs. Cry** | **Polystyrene** | **4** | **BEB-029** | **0.408** | **0.188** | **0.166** |
|  |  |  | **BEB-295** | **0.633** | **0.027** | **0.400** |
|  |  |  | **SH1000** | **-0.164** | **0.610** | **0.027** |
|  |  | **18** | **BEB-029** | **0.906** | **<0.001** | **0.820** |
|  |  |  | **BEB-295** | **0.741** | **0.006** | **0.550** |
|  |  |  | **SH1000** | **0.742** | **0.006** | **0.551** |
| **Cry vs. CFU** | **Polystyrene** | **4** | **BEB-029** | **0.628** | **0.029** | **0.395** |
|  |  |  | **BEB-295** | **0.202** | **0.529** | **0.041** |
|  |  |  | **SH1000** | **0.153** | **0.636** | **0.023** |
|  |  | **18** | **BEB-029** | **0.323** | **0.306** | **0.104** |
|  |  |  | **BEB-295** | **-0.041** | **0.899** | **0.002** |
|  |  |  | **SH1000** | **0.274** | **0.389** | **0.075** |
